# Challenges in initiating antiretroviral therapy for all HIV-infected people regardless of CD4 cell count

**DOI:** 10.1186/s40249-016-0179-9

**Published:** 2016-09-05

**Authors:** Jean Joel R. Bigna, Claudia S. Plottel, Sinata Koulla-Shiro

**Affiliations:** 1 Department of Epidemiology and Public Health, Centre Pasteur of Cameroon, 451, Rue 2005, P.O. Box 1274, Yaounde, Cameroon; 2 Bordeaux School of Public Health, University of Bordeaux, Bordeaux, France; 3 Department of Medicine, Division of Translational Medicine, New York University Langone Medical Center, New York, NY USA; 4 Faculty of Medicine and Biomedical Sciences, University of Yaounde 1, Yaoundé, Cameroon; 5 Infectious Diseases Unit, Yaounde Central Hospital, Yaounde, Cameroon

**Keywords:** Challenges, CD4 count, Resource limited setting, HIV, WHO guidelines, Early initiation, Immediate initiation, Antiretroviral, Universal access

## Abstract

**Introduction:**

Recently published large randomized controlled trials, START, TEMPRANO and HPTN 052 show the clinical benefit of early initiation of antiretroviral treatment (ART) in HIV-infected persons and in reducing HIV transmission. The trials influenced the World Health Organization (WHO) decision to issue updated recommendations to prescribe ART to all individuals living with HIV, irrespective of age and CD4 cell count.

**Discussion:**

It is clear that the new 2015 WHO recommendations if followed, will change the face of the HIV epidemic and probably curb its burden over time. Implementation however, requires that health systems, especially those in low and middle-income settings, be ready to face this challenge on a large scale. HIV prevention and treatment are easy in theory yet hard in practice. The new WHO guidelines for initiation of ART regardless of CD4 cell count will lead to upfront increases in the costs of healthcare delivery as the goal is to treat all those now newly eligible for ART. Around 22 million people living with HIV qualify and will therefore require ART. Related challenges immediately follow: firstly, that everyone must be tested for HIV; secondly, that anyone who has had an HIV test should know their result and understand its significance; and, thirdly, that every person identified as HIV-positive should receive and remain on ART. The emergence of HIV drug resistant strains when treatment is started at higher CD4 cell count thresholds is a further concern as persons on HIV treatment for longer periods of time are at increased risk of intermittent medication adherence.

**Conclusions:**

The new WHO recommendations for ART are welcome, but lacking as they fail to consider meaningful solutions to the challenges inherent to implementation. They fail to incorporate actual strategies on how to disseminate and adopt these far-reaching guidelines, especially in sub-Saharan Africa, an area with weak healthcare infrastructures. Well-designed, high-quality research is needed to assess the feasibility, safety, acceptability, impact, and cost of innovations such as the universal voluntary testing and immediate treatment approaches, and broad consultation must address community, human rights, ethical, and political concerns.

**Electronic supplementary material:**

The online version of this article (doi:10.1186/s40249-016-0179-9) contains supplementary material, which is available to authorized users.

## Multilingual abstracts

Please see Additional file 1 for translations of the abstract into the five official working languages of the United Nations.

## Background

In 2013, the World Health Organization (WHO) recommended that antiretroviral therapy (ART) should be prescribed to individuals infected with human immunodeficiency virus (HIV) whose CD4+ T-cell (CD4) counts were less than 500 cells per μL, to HIV-infected pregnant women, to HIV-discordant partners, to persons with certain medical conditions such as active tuberculosis and active hepatitis B and to individuals with severe or advanced HIV clinical disease [1]. The quality of evidence for the 2013 recommendations varies from low (pregnant women, tuberculosis and hepatitis B) to high (serodiscordant couples) related to the methodological quality of conducted studies in this field [2]. Although the debate continues about the optimal timing of ART initiation in the course of HIV infection, several lines of evidence indicate that starting ART as soon as possible in HIV patients is advantageous. In 2009, a mathematical model using a hypothetical population and available data and assumptions (including heterosexual mode of transmission) relative to a South African setting suggested that scaled-up universal voluntary HIV testing with immediate ART, combined with existing prevention approaches like circumcision, would have a major effect on severe generalized HIV epidemics, leading to a near stoppage of HIV transmission [2]. Variability in social issues between HIV epidemics is an important consideration. Indeed, any model should integrate characteristics of the sexual partner network, voluntary HIV counseling and testing uptake, linkage to care, ART coverage, and consideration of key populations [3–5]. A more recent mathematical model (also assuming heterosexual transmission) addresses the feasibility of widespread ART prescription within the context of a clinical trial conducted in Zambia and South Africa and indicates that a prevention package including home-based voluntary testing and counseling, and ART for HIV positive individuals could reduce HIV incidence by more than 60 % over 3 years [6]. It has also been demonstrated in well-conducted, large observational studies that HIV infection incidence at the community level can be reduced by early ART initiation [7, 8].

Three recently published randomized controlled trials, START [9], TEMPRANO [10] and HPTN 052 [11] have shown promising, albeit modest benefit for HIV-infected persons at an individual level, and for reducing HIV transmission following early treatment initiation. All the trials significantly informed the WHO in decision to issue the updated recommendation in September 2015 that immediate ART be initiated in all individuals diagnosed with HIV, irrespective of age and CD4 cell count [12]. The new 2015 guidelines also integrate for the first time, the provision of pre-exposure prophylactic (PrEP) interventions for all individuals at high risk of HIV infection. The recommendation for PrEP is based on clinical trial results confirming the effectiveness of tenofovir for use as PrEP to prevent people from acquiring HIV in a wide variety of settings and populations [13–15].

The Joint United Nations Program on HIV/AIDS (UNAIDS) has an ambitious goal that by 2020, 90 % of all people living with HIV should know their HIV status; 90 % of people diagnosed with HIV infection should receive sustained ART; and 90 % of all people receiving ART should have ribonucleic acid HIV suppression [16]. It is clear that if widely and successfully carried out, the new 2015 WHO recommendations will change the face of the HIV epidemic and may significantly curb its burden. However, successful adoption of the guidelines requires that health systems, particularly those in low and middle income settings, be prepared to face the challenges that implementation will bring. The aim of this paper is to present challenges of the implementation of the new WHO guidelines that is, initiating ART regardless of CD4 cell count.

## Challenges of the implementation of the new WHO guidelines

### Challenges for health systems in Africa

Table 1 summarizes challenges in meeting the new WHO guidelines. HIV prevention and treatment are easy to delineate in theory, but hard to implement in practice. The new WHO recommendations for initiation of ART regardless of CD4 cell count will incur increased costs for such treatment of all HIV-infected people [17]. Of the 37 million HIV-infected people across the globe, 70 % reside in sub-Saharan Africa, which is the most affected region. Of all HIV-infected people across the globe, only 15 million actually receive ART. Thus, we estimate that about 22 million (around 60 %) people living with HIV/acquired immunodeficiency syndrome (AIDS) will now be additional candidates for ART based on the newest WHO guidelines, and that their ART treatment should be initiated as soon as possible. Certain health care facilities in sub-Saharan Africa which will be distribution centers for ART have insufficient (or no) stock of ART. The absence of ART drugs is a major “real life” threat to the implementation of universal testing and immediate treatment. In such a situation, how will health systems in sub-Saharan Africa guarantee that ART medication be available to patients when indicated, and how will they realistically be able to meet the workload involved in adherence to the WHO recommendations and guidelines? Significant financial resources and logistical support are urgently needed to guarantee adequate supplies of ART day-to-day and to ensure the manpower for screening, counselling, and follow-up over time. Health policy makers and funders should actively engage in all aspects of relevant initiatives geared to facilitating the adoption practices that lead to compliance with the 2015 WHO mandates.

**Table 1:** Challenges for the implementation of antiretroviral therapy for all HIV-infected people regardless of CD4 cell count in Africa

Challenges for health systems in Africa
• Lack of required financial resources for HIV programs
• Increase in healthcare workers’ workload without corresponding increase in manpower
• Inadequate stocks of antiretroviral therapy
• Limited supportive health system infrastructures in resource limited settings
Challenges for universal HIV testing
• Fear of being HIV positive, fear of stigma and discrimination during home-based HIV testing
• Thoughts of not being at risk for HIV infection
• Fear of lack of privacy and confidentiality during home-based HIV testing
• Need of spouses’ for HIV home testing
• Lack of proposition of HIV testing in some health facilities
• Lack of mobile HIV testing in some settings
Challenges for linkage to care and early/immediate initiation of antiretroviral therapy
• Weak relation between mobile and home based HIV testing and health facility based antiretroviral initiation
• True refusal and false acceptance of ART initiation after HIV positive testing at home or by mobile units
• Absence of specific guidance on actual “real-life” implementation of screening, diagnosis, and initiation of antiretroviral therapy in all HIV-infected persons in resource-limited settings
Challenges for retention in care and adherence to antiretroviral treatment
• Absence of proven methods to ensure long time adherence to ART and retention in care for all HIV-infected persons in resource limited setting
• Weak early warning indicators for HIV drug resistance

### Challenges for HIV testing

Barriers for HIV testing in Africa can include no proposing for HIV testing during health consultations; thinking not at risk for HIV infection; fear of being HIV positive; fear of stigma if HIV positive; no available cure; fear of no privacy and no confidentiality, no permission from partner especially for women [18]. Strategies are needed to properly diagnose all HIV-infected people, focusing on those whose HIV status is presently unknown. Worldwide, only half of the persons with HIV infection are aware of their status [17] and in low-income and middle-income countries only 20 % know about their seropositive status [5]. Early diagnosis and timely access to care can improve the clinical course of the disease and potentially reduce transmission rates. The greatest difficulty is to know how to ensure that everyone has access to HIV testing in a weak healthcare system. If the test is available, how to ensure that everyone who has access to HIV testing be tested? How to get people to go to HIV clinics for testing? On the other hand, what are the ways and methods that can be used to reach all the people by going to them for HIV testing? While systematic HIV testing can be used in primary health care setting [19], another approach would be to conduct a community-based integrated prevention campaign [20], national HIV voluntary counseling and testing campaign [21], or mobile HIV testing [22–26]. Throughout different regions in Africa with different cultures, researches are needed to identify specific determinants related to non-acceptance of voluntary HIV counseling and testing [27–30]. This will help understand why some people do not accept HIV testing.

HIV can be diagnosed both in symptomatic and in asymptomatic individuals. Among those who are asymptomatic, HIV voluntary counseling and testing is the first step to introducing people to effective HIV/AIDS care, and is a focal point of HIV prevention in developing countries [31–33]. The facts highlight several crucial challenges: firstly, that everyone must be tested for HIV; secondly, that anyone who has had HIV testing should know their result and understand its significance; thirdly, that a person identified as HIV-positive should receive and remain on ART. Expanding universal HIV testing will require innovative approaches such as home-based HIV counseling and testing [34, 35]. There is recent evidence from two randomized controlled trials that home-based voluntary HIV counseling and testing increases the overall rate of HIV testing and an individual’s knowledge of their HIV status, when compared to health care facility-based HIV interventions [36–38]. The generalizability of findings from such randomized controlled trials to “real world” settings remains undetermined.

### Challenges for linkage to care and early/immediate initiation of antiretroviral therapy

While definitions vary concerning linkage to care [39, 40], the most important endpoint is now immediate initiation of ART regarding new 2015 WHO guidelines [12]. Home-based interventions are a necessary first step to introduce increased ART coverage to eligible patients. The next steps include the prescription of indicated medication, and the long-term adherence to that treatment. Before this, it is necessary that newly infected people link to health facilities for ART initiation. A call center can be used to increase linkage to care [39]. Two ongoing randomized trials conducted under real conditions to assess the effectiveness at the community level of immediately initiating ART after home-based HIV counseling and testing in South-Africa and Zambia are underway [41, 42]. Immediate initiation of ART will likely prove burdensome in terms of both cost and manpower for health systems in sub-Saharan Africa and for HIV-infected patients.

Some HIV-infected people encounter obstacles in accessing necessary HIV/AIDS care once their home-based diagnosis is made when they receive a referral to a health facility for the purpose of ART initiation and follow-up. A recent South African study indicated that only 12 % of people receiving an HIV diagnosis through home-based diagnostic testing were linked to HIV/AIDS care, including ART [37]. Innovative approaches implementing effective strategies for screening, testing, and maintaining HIV-infected patients on ART are therefore necessary to address such gaps in developing countries. Identification and implementation of relevant initiatives are also challenges in their own right and although they may differ from country to country, represent an urgent public health imperative. Mobile units for example, have shown their positive performance in increasing HIV screening, retention in HIV treatment, and adherence to ART [22–26], one of such barriers is ART refusal, a phenomenon in which HIV-infected individuals choose not to start treatment upon learning their ART eligibility [43]. As shown in a study, subjects which are tested in an HIV clinic were more likely to link to care and thus to initiate ART compared to subjects using HIV mobile testing [23]. However, facility based HIV counseling and testing, although important, is unlikely to be sufficient to curb the HIV epidemic since many people in sub-Saharan Africa do not have regular access to health care. As concluded in a systematic review, scaling a combination of community HIV testing and counselling, mobile testing and self-testing, with regard to the local epidemic setting, is crucial to achieve high knowledge of HIV status and linkage to HIV treatment and prevention in sub-Saharan Africa [40]. The next logical step after testing for HIV using that novel approach would be the prescription of treatment via these mobile units or home-based teams. Unfortunately these strategies have not been rigorously assessed scientifically or validated in the field. Targeted strategies will furthermore need to be implemented to diagnose and treat currently underserved and difficult-to-reach populations, such as men who have sex with men, sex workers, injecting drug users, and transgender individuals.

### Challenges for retention in care and adherence to antiretroviral treatment

The emergence of drug resistant HIV strains is a concern in situations where ART is started early [43, 44], as in treatment regimens where of ART is initiated irrespective of CD4 cell counts, since people will be on HIV treatment for longer with increased potential for of sub-optimal (intermittent) adherence. One hypothesis is that some HIV-infected people with early/immediate ART initiation, may feel well and perceive themselves as “healthy” and consequently take ART intermittently, placing them at risk for the emergence of ART resistance or become lost to follow-up. However, this should not slow down the early/immediate ART initiation once the HIV diagnosis is done since several studies reported that ART initiation at high CD4 cell count was associated with low HIV drug resistance [45, 46]. Long duration randomized controlled trials are needed to confirm findings of observational studies. Although the limited available data do not conclusively suggest an increased risk of drug resistance with earlier HIV treatment, it remains a concern and health care systems should consider measures to encourage long-term adherence. The immediate initiation of ART itself along with better coverage of all people living with HIV, might lead to a “normalization” and greater acceptance of ART. This “normalization” could reduce the stigmatization of people living with HIV and contribute to improved treatment adherence rates.

To measure the resistance to ART, the WHO recommends that health systems use early warning indicators for HIV drug resistance [47]. These indicators include good ART prescribing practices, low rate of patients lost to follow-up, high rate of patient retention on first line ART; a good on-time drug pick-up, and continuous drug supply. These indicators are part of the continuum of HIV infection control (testing, linkage, retention, treatment, and adherence) which are necessary to achieve the UNAIDS 90-90-90 target [16]. Studies demonstrated that in Africa some of these early warning indicators for HIV drug resistance are very weak [48–52]. How can these weak health systems fill out WHO and UNAIDS goals?

## Conclusions

The 2015 WHO recommendations regarding the broad prescription of ART in all HIV-infected individuals have a rational basis and are scientifically sound, yet, they are also to a large extent challenging. Compliance with the guidelines will lead to upfront increases in the costs of healthcare delivery given the goal of treating all those now newly eligible for ART, a number that approaches 22 million people living with HIV. Lacking is guidance as to actual strategies on how to disseminate and implement the recommendations at the community level, especially in sub-Saharan Africa, a region with a heavy burden of HIV and AIDS and weak health care systems. High quality research to assess the feasibility, acceptability, impact, safety, and cost of innovations such as universal voluntary testing and immediate treatment in real world settings is crucial at this juncture in time, and broad collaborations are urgently needed to address community, human rights, ethical, and political concerns. Discussion is also needed on ART provision for HIV infected people during epidemics of varying intensity. WHO has committed to promoting consultation among countries and stakeholders about the pressing biomedical and political issues of ART for HIV prevention regardless of CD4cell counts. We fervently hope that the commitment will yield tangible and practical guidance along with targeted funding to help bring the recommended treatment to those who need it most.

## Abbreviations

AIDS, acquired immunodeficiency syndrome; ART, antiretroviral therapy, also antiretroviral treatment; HIV, human immunodeficiency virus; PrEP, pre-exposure prophylactic; UNAIDS, Joint United Nations Program on HIV/AIDS; WHO, World Health Organization

## Additional file

Additional file 1: Multilingual abstracts in the five official working languages of the United Nations. (PDF 305 kb)
