# Supplementary material for: Challenges in initiating antiretroviral therapy for all HIV-infected people regardless of CD4 cell count
Source: Infect Dis Poverty. 2016 Sep 5;5(1):85. doi: 10.1186/s40249-016-0179-9 (PMC5011352; doi:10.1186/s40249-016-0179-9)
Supplement: Additional file 1: — Multilingual abstracts in the five official working languages of the United Nations. (PDF 305 kb) [file 40249_2016_179_MOESM1_ESM.pdf]

Translation of the abstract into the six official working languages of the United Nations

التحديات في بدء العلاج بمضادات الفيروسات القهقرية لجميع المصابين بفيروس نقص المناعة المكتسبة بغض النظر عن عدد خلايا CD4

جين حويل بيغنا- كلوديا بلوتيل- سيناتا كوللا شيرو.
Jean Joel R. Bigna, Claudia S. Plottel, and Sinata Koulla-Shiro

ملخص الدراسة
المقدمة:
أظهر عدد كبير من التجارب العشوائية التي تم نشرها مؤخراً START, TEMPRANO and HPTN 052 فوائد البدأ المبكر بعلاج المصابين بمرض نقص المناعة المكتسبة بعلاج مضادات الفيروسات القهقرية والحد من انتقاله. حيث أن هذه التجارب أثرت على قرار منظمة الصحة العالمية باصدار تحديث لتوصياتها لتقديم العلاج بالفيروسات القهقرية كوصفة طبية لكل المصابين بمرض نقص المناعة المكتسبة بغض النظر عن عمر المريض أو عدد خلايا CD4.
المناقشة: من الواضح أن توصيات عام 2015 ان تم تطبيقها ستغير وجه وباء فيروس نقص المناعة المكتسبة وقد تحد من عبئه على مر الزمن. الا أن تطبيق هذه التوصيات يتطلب ان يكون النظام الصحي خصوصا الأنظمة ذات الدخل المنخفض او المتوسط على استعداد لمواجهة هذا التحدي على نطاق واسع. حيث أن الحد وعلاج مرض فيروس نقص المناعة المكتسبة سهل من الناحية النظرية الا أن تطبيقه صعب من الناحية العملية. ستؤدي مبادئ وتوجيهات منظمة الصحة العالمية للبدأ بالعلاج بمضادات الفيروسات القهقرية بغض النظر عن عدد خلايا CD4 الى زيادة في تكاليف الرعاية الصحية اذا أن الهدف هو تقديم العلاج لجميع المؤهلين للحصول على العلاج بمضادات الفيروسات القهقرية.هناك حوالي 22 مليون شخص مصابين بفيروس نقص المناعة المكتسبة وبالتالي هم مؤهلين للحصول على العلاج بمضادات الفيروسات القهقرية. أولا: يجب أن يتم اجراء اختبار فيروس نقص المناعة المكتسبة للجميع. ثانيا: يجب على كل شخص مصاب بفيروس نقص المناعة المكتسبة أن يعرف النتئج ومغزاها. ثالثا: كل شخص مشخص على أنه مصاب بفيروس نقص المناعة المكتسبة يجب أن يتلقى ويستمر بالعلاج بمضادات الفيروسات القهقرية. ان ظهور سلالات مقاومة للدواء المستخدم لعلاج فيروس نقص المناعة الكتسبة عند بدء العلاج في عتبات عدد مرتفع لخلايا CD4 هو مصدر قلق إضافي اذ أن الأشخاص المصابين بفيروس نقص المناعة المكتسبة ويتلقون العلاج لفترات طويلة هم معرضون لمخاطر الالتزام بالعلاج لفترات متقطعة.
الخاتمة: ان توصيات منظمة الصحة العالمية لستخدام مضادات الفيروسات القهقرية هي موضع ترحيب الا أنها تفتقر للحلول المجدية لمواجهة التحديات التي ينطوي عليها تنفيذ هذه التوصيات. حيث أن هذه التوصيات تفتقر لدمج استراتيجيات فعلية حول كيفية نشر واعتماد هذه المبادئ بعيدة المدى ولا سيما في أفريقيا جنوب الصحراء الكبرى وهي منطقة تعاني من ضعف البنية التحتية والرعاية الصحية. ولهذا فان هناك حاجة لبحوث علمية مصممة تصميماً جيداً وذات جودة عالية لتقييم امكانية وأمان والقبول والأثر وتكلفة الابتكارات مثل الاختبار العالمي الطوعي ومنهجية العلاج الفوري. ويجب ان يتواجد مشاورات واسعة لمعالجة القلق الانساني والأخلاقي والسياسي لهذه الابتكارات..

Translated from English version into Arabic by Randa82, through

TRANSLATORS
WITHOUT BORDERS

不计 CD4 细胞数目对所有艾滋病病毒感染者启动抗逆转录病毒疗法的挑战

Jean Joel R. Bigna, Claudia S. Plottel, and Sinata Koulla-Shiro

摘要
简介：最近发表的大型随机对照试验（START、TEMPRANO 和 HPTN 052）研究显示，对艾滋病病毒感染者早期启动抗逆转录病毒治疗（ART）显示出临床效益，并能减少艾滋病传播。这些试验影响了世界卫生组织（WHO）的有关决定：不论艾滋病病毒感染者年龄和 CD4 细胞数目，最新建议是所有感染者启动抗逆转录病毒治疗。
讨论：很显然，如果遵循 2015 年 WHO 最新的建议，艾滋病流行情况将会改善，随着时间的推移很可能会减轻其疾病负担。然而，特别是低、中收入国家或地区的卫生体系，随时准备面对这一大规模挑战。在理论上艾滋病预防和治疗是很容易的，但是很难实践。由于我们的目标是现在所有新适合抗逆转录病毒治疗的感染者均进行治疗，因此不计 CD4 细胞数目对艾滋病病毒感染者早期启动该治疗方式的最新世卫组织的准则将导致医疗服务的预付费用增加。约 22 万艾滋病病毒感染者有资格进行抗逆转录病毒治疗，因此需要该治疗方法。相关的挑战紧随其后。首先，每个人均需进行艾滋病病毒检测；其次，做过艾滋病病毒检测的任何人均应该知道自己的检测结果，并理解其意义；第三，每个确诊为 HIV 阳性者均应接受并维持抗逆转录病毒治疗。 在治疗过程中，当达到较高的 CD4 细胞计数阈值时，开始出现 HIV 耐药株，与 HIV 治疗时间较长者相比，间歇服药的风险增加，更需得到关注。
结论：WHO 关于抗逆转录病毒治疗的建议是值得赞许的，但不足之处在于他们没有考虑到在具体实施中的解决方案所面临的挑战。他们没有制定如何传播和采纳这些影响深远指引的实际策略，尤其是在基础设施薄弱及医疗保健的撒哈拉以南非洲地区。我们需要精心设计的，高质量的研究来评估该治疗方法的可行性、安全性、可接受性、影响力和创新成本。如普遍自愿检测和及时的治疗方法，并广泛协商关注解决社会、人权、伦理和政治问题。

Translated from English version into Chinese by Lei Sun, and edited by Yang Pin

CDC
CHINESE CENTER FOR DISEASE CONTROL AND PREVENTION
NATIONAL INSTITUTE OF PARASITIC DISEASES

Difficultés de la mise en place d’un traitement antirétroviral contre le VIH pour tous les patients séropositifs, quelle que soit leur numération de CD4

Jean-Joël R. Bigna, Claudia S. Plottel et Sinata Koulla-Shiro

Résumé
Introduction: Les grands essais contrôlés randomisés START, TEMPRANO et HPTN 052, publiés récemment, démontrent l'intérêt clinique de la mise en place précoce du traitement antirétroviral (TAR) chez les patients infectés par le VIH pour réduire la transmission du virus. Ces essais ont pesé sur la décision de l'OMS de publier des recommandations actualisées préconisant la prescription d'un TAR à tous les patients séropositifs au VIH, quels que soient leur âge et leur numération de CD4.
Discussion: Il est évident que les nouvelles recommandations émises par l'OMS en 2015 pourraient changer la face de l'épidémie de VIH et probablement, à terme, en réduire le fardeau si elles sont suivies. Cependant, il faudra pour cela que les systèmes de santé publique soient prêts à relever le défi à grande échelle, en particulier dans les pays à bas et moyens revenus. La prévention et le traitement du SIDA sont faciles en théorie mais difficiles en pratique. Les nouvelles lignes directrices de l'OMS concernant la mise en place d'un TAR quelle que soit la numération de CD4 vont entraîner, dans un premier temps, une hausse des frais de santé puisque le but est de traiter tous les patients qui deviennent ainsi éligibles. Cela signifie que quelque 22 millions de séropositifs vont avoir droit à un TAR. Il en découle un certain nombre de difficultés immédiates : pour commencer, tout le monde doit faire l'objet d'un test de dépistage du VIH ; deuxièmement, tous ceux qui ont été dépistés doivent connaître le résultat du test et comprendre ses implications ; troisièmement, chaque personne identifiée comme séropositive doit recevoir un TAR et le continuer. L'émergence de souches du VIH résistantes au traitement si celui-ci est engagé à partir d'un seuil élevé de CD4 pose encore un autre problème car les personnes traitées depuis longtemps sont les plus susceptibles de « décrocher » de temps en temps de leur traitement.
Conclusions: Les nouvelles recommandations de l'OMS concernant le TAR sont les bienvenues mais elles ne proposent pas de solutions pertinentes aux problèmes que soulève leur mise en application. Leurs instructions ont une portée très vaste mais n'intègrent pas de stratégies réelles pour leur diffusion et leur adoption, en particulier dans les pays d'Afrique subsaharienne où les infrastructures de santé sont peu développées. Des recherches bien conçues et de bonne qualité sont nécessaires pour évaluer la faisabilité, l'innocuité, l'acceptabilité, l'impact et le coût d'innovations telles que le dépistage volontaire universel et le traitement immédiat. Une large consultation doit être engagée afin d'examiner les questions communautaires, éthiques, politiques et de droits humains.

Translated from English version into French by Suzanne Assenat, through

Проблемы внедрения антиретровирусной терапии для всех ВИЧ-инфицированных вне зависимости от количества клеток CD4

Жан Жоель Р. Бинья, Клаудия С. Плоттел и Сината Кулла-Широ

Аннотация
Введение: Недавно опубликованные крупномасштабные рандомизированные контролируемые исследования, такие как START, TEMPRANO и HPTN 052, продемонстрировали улучшение клинических результатов, связанных с досрочным проведением антиретровирусной терапии (АТР) у ВИЧ-инфицированных, а также сокращение случаев ВИЧ-инфицирования. Данные исследования способствовали тому, что Всемирная организация здравоохранения (ВОЗ) приняла решение об издании обновленных рекомендаций в отношении АТР для всех людей, живущих с диагнозом ВИЧ, независимо от их возраста и количества клеток CD4.
Исследование: Очевидно, что если соблюдать новые рекомендации ВОЗ от 2015 г., то это изменит лицо ВИЧ эпидемии, а также сдержит ее распространение. Однако, чтобы использовать эту терапию, необходимо, чтобы системы здравоохранения, особенно находящиеся в малоимущих странах, были готовы столкнуться с этой проблемой на достойном уровне. В теории профилактики и лечения ВИЧ нет ничего сложного, однако на практике все не так просто. Новые рекомендации ВОЗ в отношении внедрения АТР вне зависимости от количества клеток CD4 приведут к предварительному повышению стоимости медицинских услуг, так как главной целью является лечение всех тех людей, которые могут проходить АТР. Около 22 миллионов людей, живущих с диагнозом ВИЧ, получат право на АТР, а, следовательно, потребуют ее прохождения. За этим незамедлительно последуют похожие проблемы: во-первых, всем нужно будет сдать анализ на ВИЧ, во-вторых, все, кто сдадут анализ, должны будут узнать о своих результатах и оценить их важность, и в-третьих, каждый ВИЧ-положительный человек должен будет получить и проходить курс АТР. Возникновение штамма ВИЧ, резистентному к лекарственным средствам, при начале лечения с высоким количеством клеток CD4, также является поводом для волнения, так как люди, проходящие длительное лечение ВИЧ, находятся в зоне повышенного риска прерывистой приверженности лечения.
Выводы: Принятие новых рекомендаций в отношении АТР приветствуется, однако не предлагает продуманных решений проблем, связанных с внедрением терапии. Они не учитывают реального положения дел по проблемам распространения и использования терапии, особенно в странах Африки к югу от Сахары, территории с плохо развитой инфраструктурой здравоохранения. Необходимо провести хорошо продуманное и высококачественное исследование, чтобы оценить выполнимость, безопасность, приемлемость, влияние и стоимость таких нововведений, как добровольное тестирование, первая медицинская помощь, а также широкие консультации, которые должны быть направлены на решение проблем общества и учитывать его этические и политические взгляды, а также права человека.

Translated from English version into Russian by Yuliya Maiseyenka, through

TRANSLATORS
WITHOUT BORDERS

Desafíos para iniciar la terapia antirretroviral para todas las personas infectadas de HIV con independencia del recuento de células CD4

Jean Joel R. Bigna, Claudia S. Plottel y Sinata Koulla-Shiro

Resumen

Introducción: En grandes ensayos controlados aleatorios recientemente publicados, START, TEMPRANO y HPTN 052, muestran los beneficios clínicos que aporta el inicio en fase temprana del tratamiento antirretroviral (TAR) en personas infectadas de HIV así como para reducir la transmisión del HIV. Los ensayos influyeron sobre la decisión de la Organización Mundial de la Salud (OMS) de actualizar sus recomendaciones para prescribir el TAR a todas las personas que viven con HIV, con independencia de su edad y recuento de células CD4.

Aspectos a tener en cuenta: Está claro que si se siguieran las nuevas recomendaciones de la OMS en 2015 cambiaría la cara de la epidemia de HIV y probablemente frenaría su avance a lo largo del tiempo. Sin embargo, su implementación exige que los sistemas sanitarios, especialmente en países con ingresos bajos y medios, estén preparados para afrontar este desafío a gran escala. La prevención y el tratamiento del HIV son fáciles en teoría pero son complicados en la práctica. Las nuevas directrices de la OMS para el inicio del TAR con independencia del recuento de células CD4 conllevará el aumento de los costes de la asistencia sanitaria ya que el objetivo es tratar a todos los nuevos candidatos para el TAR. Unos 22 millones de personas que viven con el HIV cumplen los requisitos y por tanto necesitarán el TAR. Éstos son los desafíos: en primer lugar, que todo el mundo sea sometido a un análisis para comprobar si tiene HIV; en segundo lugar, que todo aquel que haya sido sometido a una prueba de HIV debería conocer su resultado y comprender su significado; y en tercer lugar, que toda persona identificada como positiva en HIV reciba y permanezca en el TAR. La aparición de cepas resistentes a los fármacos para HIV cuando se inicia el tratamiento con un recuento más elevado de células CD4 aumenta la preocupación ya que las personas sometidas a tratamiento para HIV durante períodos más largos de tiempo tienen un mayor riesgo de adherencia intermitente a la medicación.

Conclusiones: Las nuevas recomendaciones de la OMS para el TAR son bienvenidas, pero insuficientes ya que no contemplan soluciones prácticas para los desafíos inherentes a su implementación. No logran incorporar estrategias reales acerca de cómo diseminar y adoptar estas directrices de gran alcance, especialmente en el África subsahariana, una zona cuya infraestructura sanitaria es muy débil. Se necesita una investigación bien diseñada y de alta calidad para evaluar la viabilidad, seguridad, aceptación, impacto y coste de innovaciones como pruebas voluntarias universales y estrategias de tratamiento inmediato, así como un amplio proceso de consulta que debe tener en cuenta a la comunidad, los derechos humanos, así como cuestiones éticas y políticas.

Translated from English version into Spanish by SergioLorenzi, through

TRANSLATORS
WITHOUT BORDERS
